# Grsf1-Induced Translation of the SNARE Protein Use1 Is Required for Expansion of the Erythroid Compartment

**DOI:** 10.1371/journal.pone.0104631

**Published:** 2014-09-03

**Authors:** Andrzej Nieradka, Christoph Ufer, Klaske Thiadens, Godfrey Grech, Rastislav Horos, Marleen van Coevorden-Hameete, Emile van den Akker, Sajad Sofi, Hartmut Kuhn, Marieke von Lindern

**Affiliations:** 1 Dept. Hematology, Erasmus MC, Rotterdam, The Netherlands; 2 Inst. Biochemistry, University Medicine Berlin-Charité, Berlin, Germany; 3 Dept. Hematopoiesis at Sanquin Research, and Landsteiner Laboratory AMC/UvA, Amsterdam, The Netherlands; 4 Dept. Pathology, University of Malta Medical School, Msida, Malta; Emory University, United States of America

## Abstract

Induction of cell proliferation requires a concomitant increase in the synthesis of glycosylated lipids and membrane proteins, which is dependent on ER-Golgi protein transport by CopII-coated vesicles. In this process, retrograde transport of ER resident proteins from the Golgi is crucial to maintain ER integrity, and allows for anterograde transport to continue. We previously showed that expression of the CopI specific SNARE protein Use1 (Unusual SNARE in the ER 1) is tightly regulated by eIF4E-dependent translation initiation of *Use1* mRNA. Here we investigate the mechanism that controls *Use1* mRNA translation. The 5′UTR of mouse *Use1* contains a 156 nt alternatively spliced intron. The non-spliced form is the predominantly translated mRNA. The alternatively spliced sequence contains G-repeats that bind the RNA-binding protein G-rich sequence binding factor 1 (Grsf1) in RNA band shift assays. The presence of these G-repeats rendered translation of reporter constructs dependent on the Grsf1 concentration. Down regulation of either *Grsf1* or *Use1* abrogated expansion of erythroblasts. The 5′UTR of human *Use1* lacks the splice donor site, but contains an additional upstream open reading frame in close proximity of the translation start site. Similar to mouse *Use1*, also the human 5′UTR contains G-repeats in front of the start codon. In conclusion, Grsf1 controls translation of the SNARE protein Use1, possibly by positioning the 40S ribosomal subunit and associated translation factors in front of the translation start site.

## Introduction

Erythropoiesis, the daily generation of large numbers of erythrocytes, requires tight control of expansion and differentiation of the progenitor compartment. This depends on the cooperation of erythropoietin (Epo) with stem cell factor (SCF) and glucocorticoids. [4], [12], [22], [55]. SCF does not exclusively act on erythroblasts, it is also important to maintain other progenitor compartments [13], and aberrant SCF signaling is observed in cancer [7], [31], [38], [57].

SCF-induced expansion of the erythroblast compartment requires activation of mTOR (mammalian target of rapamycin) and subsequent release of eIF4E (eukaryote translation initiation factor 4E) [10], [24]. The availability of eIF4E regulates the rate of mRNA translation in general, but transcripts with a long and structured 5′UTR are hypersensitive to eIF4E levels, whereas transcripts with a short and simple 5′UTR are only mildly affected by eIF4E levels [26], [33]. We previously identified transcripts that are hypersensitive to eIF4E levels for their translation [24]. They included *Use1* (unusual SNARE in the ER-1), the mammalian homologue of the yeast SNARE (Soluble N-ethylmaleimide-sensitive factor attachment protein (SNAP) receptor) *Use1p* [24], [54].

Mammalian Use1 is also known as mEd2, as MAPK-activating protein PM26 [39], as p31 [50], and as Q-SNARE D12 [42]. Use1 is involved in retrograde transport of CopI coatomer coated vesicles and forms a complex with t-SNARE proteins syntaxin18 (Ufe1p) and Bnip1 (Sec20p) in the ER. This t-SNARE complex fuses with v-SNARE protein Sec22b (Sec22p) present on vesicles transporting ER proteins from the Ergic (ER-Golgi intermediate compartment) and cis-Golgi back to the ER [8], [16], [21]. Mice lacking Use1 die during embryogenesis before E8.5, and deletion of *Use1* in MEFs (mouse embryo fibroblasts) causes a lethal disruption of ER structures [50].

Retrograde transport by CopI vesicles shuttles chaperones and other ER-resident proteins back from the Golgi to the ER, which is a requirement for anterograde transport of lipids and proteins to the Golgi by CopII vesicles [11], [34]. Anterograde ER to Golgi transport depends, among others, on Sar1, member of the Arf family of GTPases, and on Sec23 as the Sar1-GAP protein [9]. Interestingly, mutations in Sec23B were identified as the genetic cause of congenital dyserythropoietic anemia type II (CDA-II) [25] and its specific erythroid phenotype is caused by cell type specific differential regulation of SEC family protein expression [48]. Obviously, the rate of anterograde transport to the Golgi has to increase in proliferating cells. SCF-induced, PI3K-dependent translation of *Use1* is a novel mechanism rendering the Golgi-coupled secretary pathway dependent on mitogenic signaling [24].

RNA-binding proteins can coordinate the translation of mRNAs encoding proteins that function in a specific pathway or in the developmental stage of a particular tissue. Misregulation of translation can cause severe diseases as exemplified by deregulation of Fragile X proteins [36]. Recently, a mitochondrial isoform of G-Rich Sequence binding Factor 1 (Grsf1) was shown to be important for the coordinated expression of mitochondrial proteins [2], [29]. Grsf1 was initially shown to bind G-rich elements present in the 5′UTRs of *influenza virus* and *glutathione peroxidase 4* (*Gpx4*) to enhance translation of these transcripts [30], [43], [51]. In mitochondria, Grsf1 cooperates with RNaseP to stabilize and translate target transcripts [29]. Proteomic analysis indicated that Grsf1 is predominantly present as a monomeric protein [2].

To investigate the mechanisms by which mRNA translation contributes to adjust expression levels of *Use1*, we identified the full 5′UTR and potential regulatory elements. A G-rich sequence located just upstream of the AUG start site is the docking site for Grsf1. Similar G-rich stretches are present just in front of the AUG start codon in human *Use1*. Modulation of Grsf1 expression directly correlated with Use1 protein expression, and both Use1 and Grsf1 appeared to be required to inhibit differentiation and sustain renewal divisions of erythroblasts.

## Materials and Methods

### Cell culture

HEK-293T and NIH3T3 cells were cultured in DMEM (Invitrogen, Breda, The Netherlands) supplemented with 10% FCS, 100 IU/ml penicillin and 100 µg/ml streptomycin. Mouse liver was isolated from a C57/B6 mouse 8 weeks of age. The I/11 erythroblast line was cultured in StemPro-34 medium (Life Technologies) as described previously [55]. For expansion, the medium was supplemented with 0.5 U/ml Epo (Ortho-Biotech, Tilburg, The Netherlands), 100 ng/ml SCF (supernatant of CHO producer cells), and 10^−6^ M dexamethasone (Dex; Sigma-Aldrich). Differentiation was induced in medium containing 2 U/ml Epo, 0.5 mg/ml holo-transferrin. Cell numbers and cell size distribution were determined using an electronic cell counter (CASY-1; Roche). Cell morphology was analyzed in cytospines stained with histological dyes and neutral benzidine [28], using an OlympusBX40 microscope and OlympusDp50 CCD camera, and Viewfinder Lite 1.0 acquisition software.

### Polyribosomal analysis

Polyribosomes were generated as described before [20], [28] with minor modifications. 40×10^6^ of I/11 cells were incubated with 0.1 mg/ml cycloheximide (Sigma Aldrich) for 10 min at 37°C, washed with cold PBS containing 0.1 mg/ml cycloheximide and lysed in buffer containing 10 mM Tris-HCl, 12 mM MgCl_2_, 140 mM NaCl, 0.5% Nonidet-P40, 500 U/ml RNAsin (Promega), 0.1 mg/ml cycloheximide, 20 mM dithiothreitol and SigmaFast protease inhibitor (Sigma Aldrich). After removal of nuclei (3000×g; 3 min) heparin was added to 3.75 mg/ml and lysates were centrifuged at 13000×g for 13 min. Supernatants were loaded on 10.5 ml 7–46% linear sucrose gradients containing 10 mM Tris-HCl, 12 mM MgCl_2_, 140 mM NaCl and centrifuged at 190,000×g for 3 hours. Measurement of absorbance at 254 nm, visualization and fractionation were done by Econo system (Biorad).

### RNA isolation, cDNA synthesis, RT-PCR and RACE

Total RNA from transiently transfected cells or from shRNA transduced I/11 cells was isolated using the Trizol reagent (Life Technologies) as recommended in the manufactures protocol. RNA from fractionated polyribosomes was isolated by proteinase K digestion, phenol-chloroform extraction and LiCl precipitation as described [20]. All RNA samples were treated with DNAse prior to cDNA synthesis according to manufacturer instructions. cDNA for real-time PCR (RT-PCR) was generated as described [28]. RT-PCR involved TaqMan technology (PE Applied Biosystems Model 7700 sequence detector), using the double stranded DNA-specific fluorescence dye SYBR green I to detect PCR product as previously described [32]. The amplification program consisted of 1 cycle of 50°C with 2 min hold (AmpErase UNG incubation), 1 cycle of 95°C with 10 min hold (AmpliTaq Gold Activation), 40 cycles of denaturation at 95°C for 15 s, annealing at 60°C for 30 s and extension at 72°C for 30 s. Gene specific primers were used (all primers are described in Table S1). For RACE experiment, 60 ng of purified poly(A)+ mRNA and GSP1 primer was used to synthesize cDNA at 60°C in accordance to manufacturer's protocol, using Transcriptor Reverse Transcriptase (Roche 03531317001) supplied together with the mRNA capture kit (Roche 117878960). First strand cDNA was purified using High pure purification columns (Roche 11732668001) and dA-tailed. The first PCR was performed using the GSP2 primer and the dT-linker forward primer supplied by the kit. Nested PCR was performed with the GSP3 primer and the forward primer supplied in the kit. Final products were cloned directly into pCR2.1 (Invitrogen) according to the instructions of the manufacturer. Nucleotide sequencing was carried out using a binding domain sequencing kit according to instructions from the provider (PE Biosystems). Sequencing was carried out on an ABI 310 automatic sequencer (PE Biosystems) using the M13 forward primer.

### Constructs

The mouse and human *Use1* UTR was amplified using the Expand High Fidelity PCR System (Roche), a forward primer harboring an XhoI site and T7 promoter, and a reverse primer containing a NcoI site. PCR products were ligated in pCR2.1 (Invitrogen) and pGL2-basic. The plasmids ΔSS, ΔGGGG and ΔAGGGCGGA were generated with the QuickChange Site-Direct Mutagenesis Kit (Stratagene). In the ΔSS the AAGAUGG sequence around the start codon was changed into a NcoI site: ACCAUGG. All modified vectors were confirmed by DNA sequencing. The constructs ΔIn, and ‘β-globin intron’ were generated by PCR cloning. The ΔIn was cloned as the spliced construct from mouse cDNA. The β- globin plasmid was constructed by replacing the *Use1* intron of WT with the 80 bp *β-globin* intron sequence from βIVS II (kind gift from S. Philipsen, Erasmus MC). To obtain the β-globin plasmid three separate PCR reactions were performed with overlapping primers containing *Use1* and *β-globin* intron sequence. PCR1 amplified *Use1* 5′ flanking sequence, PCR2 the mini β-globin intron, and PCR3 the 3′flanking sequence to the AUG start codon. The three PCR products were purified on agarose gel. PCR1 was fused to PCR2 product using forward primer PCR1 and reverse primer from PCR2, and PCR3 was fused using forward primer from PCR1 and reverse primer PCR3. The Grsf1 plasmid was cloned to pcDNA3.1(-) [52]. All indicated primers are shown in Table S1.

### *In vitro* transcription and translation

Reporter plasmids (pGL2-basic) were linearized downstream of the luciferase coding sequence (Sal1). *In vitro* transcription was done with the Riboprobe in vitro Transcription Systems (Promega). *In vitro* translation assay were carried out with the Rabbit Reticulocyte Lysate System (Promega), according to the manufacture's protocol. After the reaction 5 µl was taken for the luciferase assay. Luciferase activity was measured using the Steady-Glo system (Promega).

### Transfection, Western Blotting and Reporter Assay

For reporter assays, 10^7^ Ba/F3 cells were electroporated (0.28 kV, capacitance 960 µFD) with maximum 20 µg of DNA. The HEK-293T cells were transfected using the transfection kit Mirus MT-1 (Mirus Bio Coorporation) in 24-well plates according to manufacture's protocol. Reporter plasmids (WT, ΔAGGGCGGA) and Grsf1 expression vector were co-transfected in NIH3T3 cells using the transfection kit Lipofectamine 2000 (Invitrogen) in 24-well plates. The reporter harboring the human *Use1* 5′UTR, and the *Grsf1* expression vector were co-transfected in 5×10^5^ HEK-293T cells/well seeded in 12-well dishes using calcium chloride. To exclude transcriptional effects due to different promoter doses, the total plasmid concentration was kept constant by adding appropriate amounts of empty expression vector pcDNA3.1(-). Transfected cells were harvested 24 hr post-transfection, or at the time indicated. Luciferase activity was measured in cell lysates using the Steady-Glo system (Promega). Transfection efficiency was determined by co-transfecting lacZ and analyzing β-galactosidase activity using the B-Gal kit (Promega). I/11 cells transduced with shRNA were harvested after the indicated time point by the addition of ice-cold PBS. Cell lysates, SDS-PAGE and Western blotting were performed as described previously [53]. To analyze Use1 and actin protein level, equal amounts of protein were loaded on a 12.5% polyacrylamide gel. The antibodies used: anti-p31 rabbit polyclonal antibody (Synaptic Systems) and anti-actin goat (Santa Cruz, CA), secondary antibody: Goat-anti-Rabbit 800 and Donkey-anti-Goat 800 (LI-COR Biosciences). Proteins were visualized using the Odyssey Infrared Imaging Machine (LI-COR Biosciences)

### Virus production, cells transduction

The pLKO.1-puro lentiviral construct containing shRNA sequences against *Use1*, *Grsf1* and control sequence were obtained from Sigma's MISSION TRC-Mm 1.0 (Mouse) shRNA library (Table S2). HEK-293T cells were transfected with 2.5 µg of pMD2.G DNA, 7.5 µg psPAX.2 DNA and 10 µg of lentiviral viral vector as described above. Medium was harvested 24 and 48 hours following transfection, and concentrated by ultracentrifugation at 60,000 g for 2 hours. The pellet was resuspended in 150 µl of 1% BSA/PBS and incubated for 2 hours at 4°C. Lentiviruses were then used directly for titration, snap-frozen and stored at −80°C. For titration, HEK-293T cells were seeded at 10^5^ per well in 6-well plates and dilutions from 10^−4^ to 10^−8^ of lentivirus supernatant were used for transduction in presence of 8 µg/ml polybrene (Sigma Aldrich). Cells were selected for 8 days with puromycine, washed with PBS, fixed with 70% ethanol and stained with crystal violet for 1 hour. Colonies were counted by microscope at a 10× magnification. Concentrated lentiviruses with titers around 500×10^6^ TU/ml were used to transduce 4×10^6^ of I/11 cells in presence of 8 µg/ml polybrene. Cells were selected by adding puromycine 24 hours post transduction.

### RNA mobility shift assays

RNA probes were generated in the presence or absence of small amounts of Digoxigenin-11-UTP (Roche), using the T7 Megashortscript Kit (Ambion) and the primers listed in Table S1. As a template, WT or mutated ΔGGGG T7 promoter driven DNA constructs were used. *In vitro* transcription products were purified using the MEGAclear kit from Ambion. For RNA/protein-binding studies, different amounts of protein (400 ng if not stated otherwise) were incubated with 0.5–1 pmol of digoxigenin-labeled RNA probe for 20 min at 30°C in binding buffer (10 mM HEPES-NaOH at pH 7.9, 25 mM KCl, 1.5 mM EDTA, 4% glycerol, 0.25 mM DTT, 7.5 µg/µL heparin, 25 ng/µL yeast tRNA) in a reaction volume of 15 µL. Afterwards the samples were analyzed on native 5% polyacrylamide gel electrophoresis. Protein/RNA complexes were transferred to a nylon membrane, and blots were visualized using the DIG Luminescent Detection Kit (Roche). The RNA binding affinity Kd was estimated as shown previously [35], [46]. Briefly, following visualization bands (free RNA versus shifted RNA) were quantified using Biorad quantification software. Then logarithmic values of the ratio of shifted (complexed) versus free RNA were plotted as function of logarithmic values of the molar amount of protein present in the reaction. This revealed a linear correlation (y = mx+n), where m, n and r^2^ were be determined in excel software. The logarithmic value of the Kd could be quantified from deducing the molar amount of protein when log (complexed RNA/free RNA) equals zero.

### Circular dichroism (CD) spectroscopy

CD spectroscopy experiments were performed as described previously with some minor alterations [6]. A 40 nt nucleotide probe of the *Use1* 5′UTR wt sequence (5′-GUA-GCA-GGG-CGG-ACC-UCG-GAG-GGG-AAG-GAC-CUC-ACU-CAG-G-3′) was used. Briefly, RNA stocks at 20 µM in Tris-HCl, pH 7.5, in the presence or absence of 100 mM of KCl or NaCl were heated to 70°C for 5 minutes and then slowly cooled down to 10°C at a rate of 0.01 K/s. RNAs were then diluted to 5 µM and CD spectra were monitored at a Jasco J720 spectropolarimeter at 10°C using a 200 µl quartz cuvette with a path length of 1 mm using a measurement range of 320 - 220 nm, a response of 2 sec, data pitch of 0.1 nm, bandwidth of 1 nm and a scanning speed of 100 nm/min. Data were accumulated from ten scans. Presented data are representative for 3 independent experiments.

### Hemoglobinisation and flow cytometry

Hemoglobin was measured as described before [3]. Propidium Iodide positive cells were measured by flow cytometry using the LSR II (Becton-Dickinson, San Jose, CA).

## Results

### Identification of the 5′UTR of Use1

**Figure 1:**
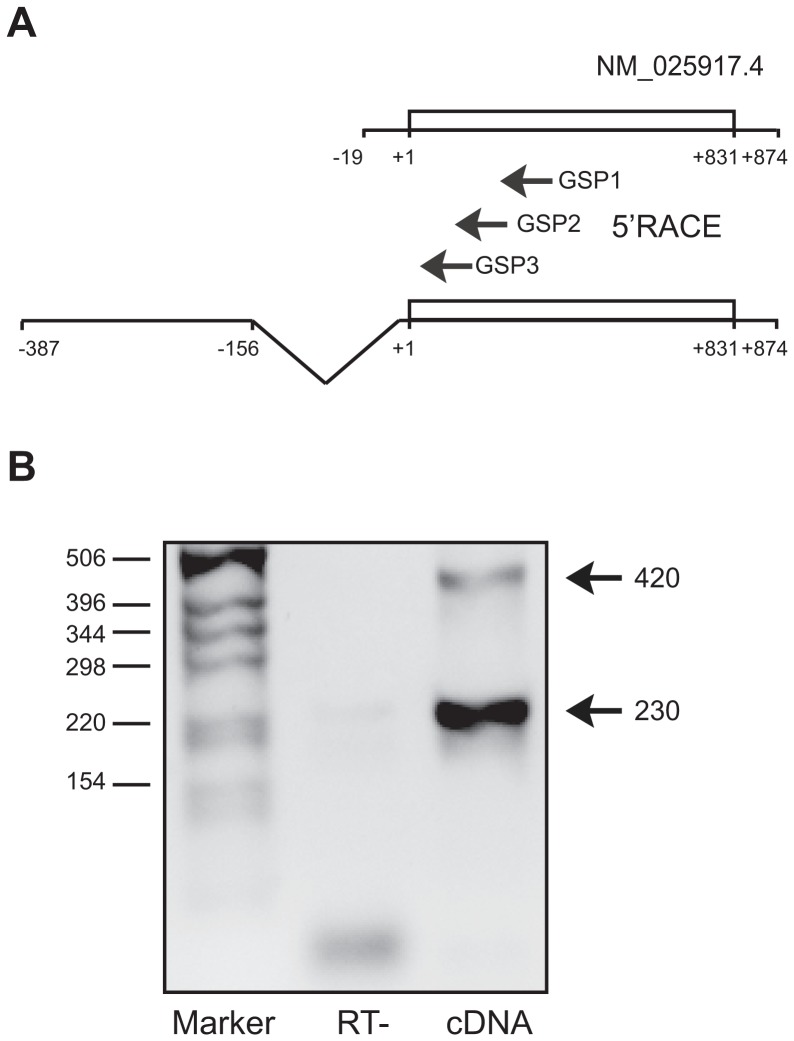
Identification of the Use1 5′UTR. (**A**) NM025917.4 represents the *Use1* cDNA sequence in Genbank. The *Use1* ORF is 831 nt long, the 3′UTR is only 43 nt long. Nested primers (GSP1-3) located downstream of the AUG start codon were used for 5′RACE which revealed a 387 nt 5′UTR with a 156 nt alternatively spliced intron just upstream of the AUG start codon (see also figure 5A). (**B**) The GSP2 primer in the 5′RACE yielded two products of 260 and 420 nt (RT-: cDNA reaction without reverse transcriptase). Fragments size is indicated in nucleotides.

To investigate polysome recruitment of *Use1* we first identified the complete 5′UTR of Use1. The reference cDNA NM_025917.4, representing *Use1* version 1, contains only a short and probably incomplete 5′UTR of 19 nt, possibly due to secondary structures in the 5′UTR that prematurely stopped the reverse transcriptase. A longer *Use1* isoform is encoded by reference cDNA NM_001145780.1 that includes an upstream exon with an alternative translation start site. For RACE (rapid amplification of cDNA ends) we used a recombinant RT-enzyme active at 60°C. Nested PCR was used in combination with primers located just downstream of the AUG start codon (figure 1A), which yielded two distinct RACE fragments, 420 and 230 nt long (figure 1B). The 420 nt fragment overlapped with EST BY097923 and represents a 5′UTR of 387 nt. The shorter fragment extends as far upstream as the long fragment, but represents a transcript in which an intron of 156 nucleotides has been spliced out exactly as represented by NM_001145780.1 (figure 1A). The alternative intron is situated just in front of the *Use1* AUG start codon.

### Efficient Use 1 expression requires intronic sequences

Alternative splicing of the 5′UTR may encode proteins with alternative start codons. In addition, splicing of the 5′UTR may affect gene expression when regulatory sequences that determine transcript stability, localization, or translation are present in the alternatively spliced intron [15], [58].

**Figure 2:**
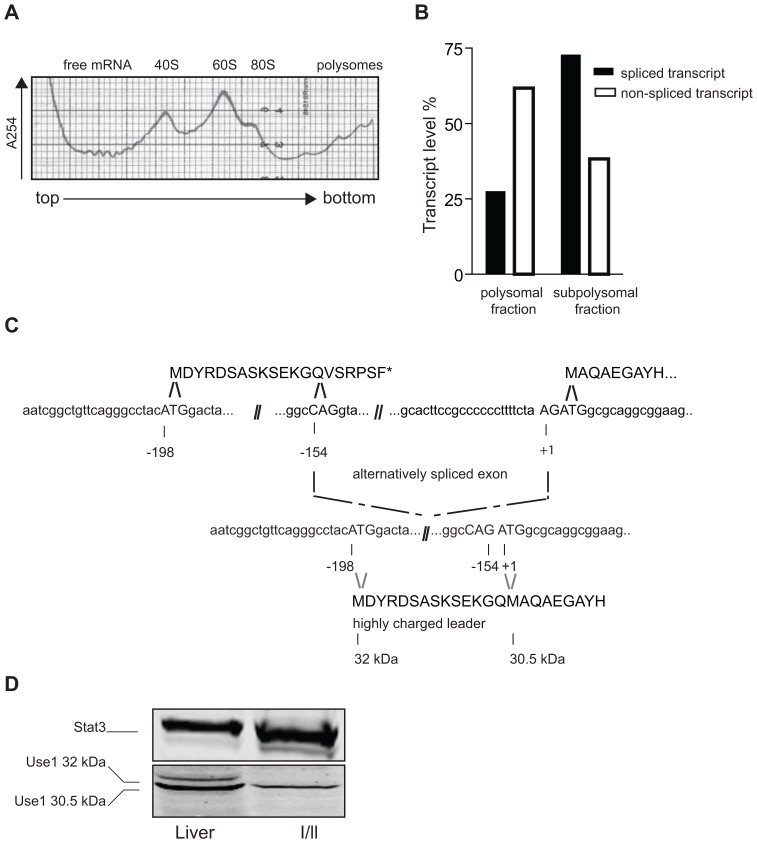
The unspliced transcript is preferentially translated. (**A**) Polysome profile of I/ll cells. Cell lysate was centrifuged on a 7–46% sucrose gradient and the distribution of RNA was measured by absorption at 254 nm. The free mRNA, ribosomal subunits and polysomes are indicated. (**B**) Real time, reverse transcriptase PCR on polysome bound (pb) and subpolysomal (sub) mRNA using a reverse primer downstream the *Use1* AUG start codon and forward primers upstream of, and within the intron to amplify the spliced (black bars) and unspliced (white bars) RNA, respectively. The percentage polysome recruitment was calculated for both transcripts. (**C**) The spliced mRNA variant encodes a longer protein variant. The top nucleotide sequence represents the unspliced transcript. At −154 and +3 the AG nucleotides that are part of the splice sites are shown in capitals. The lower nucleotide sequence represents the spliced transcript. A start codon at nucleotide −108, compliant with the Kazak consensus, appears to be in frame with the main ORF in the spliced transcript, whereas it is followed by stop codon in the intron in the unspliced transcript. Predicted protein sequences are shown above the nucleotide sequences. Slashes indicate gaps in the sequence as only important parts of the sequence are shown. (**D**) Protein expression in cultured I/11 erythroblasts and in primary liver cells (C57/B6). Western blot probed with anti-Use1, and with anti-Stat3 as loading control. Use 1 isoforms are indicated.

We isolated subpolysomal and polysome bound mRNA from erythroblasts and determined the presence of spliced and unspliced *Use1* mRNA by RT-PCR in each fraction (figure 2A,B). We found 65% of the unspliced form, and only 25% of the spliced form of *Use1* transcript present in the actively translated fraction of polyribosomes, which suggested that the presence of the intron stimulated translation initiation (figure 2A,B). Although the spliced *Use1* transcript was far more abundant than the unspliced *Use1* transcript in the 5′RACE experiment (figure 1B), RT-PCR indicated near equal expression in total RNA (data not shown).

Next we examined protein expression. A start codon 44 nt upstream of the splice donor site gives rise to a short upstream ORF (uORF) in the unspliced transcript, and to a Use1 protein with a 14 amino acid extension at the N-terminus encoded by the spliced transcript (figure 2C). On Western blot we compared Use1 expression in erythroblasts, with Use1 expression in liver, an organ with high transcript expression. Only the p31 isoform of Use1, translated from the unspliced transcript, is detected in erythroblasts (figure 2D). A small amount of the longer isform is detectable in liver cells.

**Figure 3:**
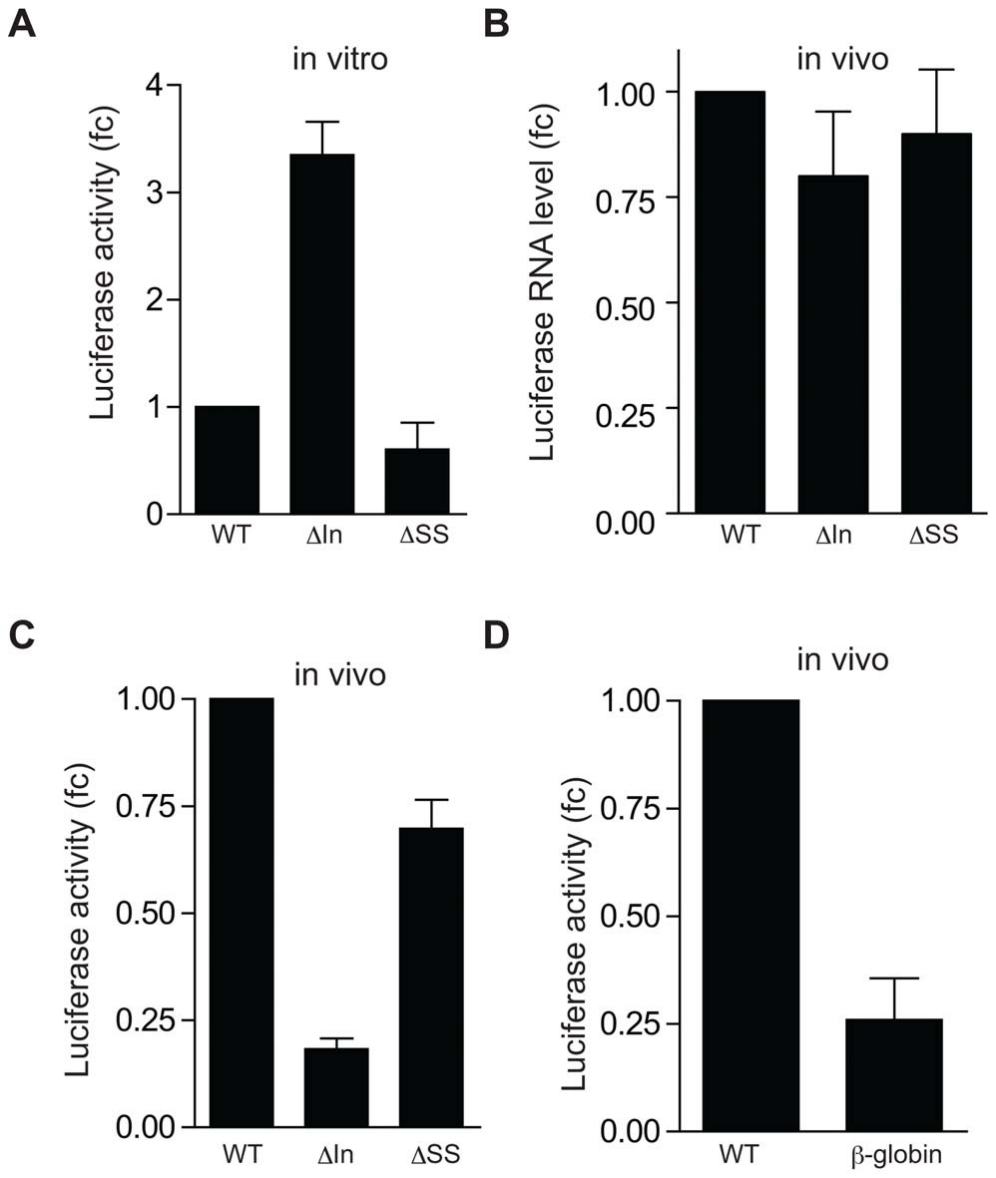
The Use1 5′ UTR controls translation of a luciferase reporter construct. (**A**) Luciferase activity (fc: fold change compared to WT) produced in reticulocyte lysate from equimolar amounts reporter RNA of reporter transcripts lacking the intron (ΔIn), or the 3′ splice site (ΔSS). (**B, C**) Luciferase mRNA expression (B) and luciferase activity (C) (fc: fold change compared to WT) in HEK-293T cell lysate following transfection of the reporter constructs used in (A). (**D**) Luciferase activity (fc: fold change compared to WT) of a reporter in which the intron is replaced by a ß-globin mini-intron. Luciferase levels are corrected for transcript expression. All bars show the average of 3 experiments, error bars indicate standard deviation.

The role of the alternative intron in *Use1* translation was further analyzed by reporter assays in which the expression of luciferase coupled to a wild type 5′UTR (WT) was compared with constructs lacking the intron (ΔIn), or containing a mutated 3′ splice site (ΔSS). First, identical molar amounts of *in vitro* synthesized mRNA were translated in reticulocyte lysate. The luciferase activity increased 3.5 fold when transcripts lacked the intron, which suggested that the intron contains sequences that inhibit *in vitro* translation (figure 3A). Deletion of the 3′ splice site did not affect *in vitro* translation (figure 3A). Next, the same reporter constructs cloned into an expression vector (pGL2 basic) were expressed in HEK-293T cells. Quantitative PCR indicated that transcript expression was similar in all experiments (figure 3B). In contrast to translation in reticulocyte lysate, luciferase activity of the transcript lacking the intron was 4-fold decreased in these cells (figure 3C). Mutation of the 3′ splice site did not drastically alter luciferase activity. The splice site overlaps the Kozak sequence around the start codon. The small reduction of luciferase activity in the ΔSS mutant may be due to the alteration of the luciferase start codon from AAG**AUG**G to ACC**AUG**G (figure 2C). We also tested a construct in which the intron was replaced by the minimal intron of the β-globin gene. Luciferase expression of this reporter construct was reduced to levels similar to those of the reporter in which the intron was deleted (figure 3D). In conclusion, the presence of the *Use1* intronic sequence enhanced translation of the endogenous transcript in erythroblasts (figure 2) and of reporter constructs transfected in HEK-293T cells (figure 3). This suggested the presence of a regulatory element in the alternatively spliced intron. Most likely, this element associates with a protein complex that is not present in reticulocyte lysate, and the secondary structure of the element may hinder *in vitro* translation resulting in reduced *in vitro* translation of transcripts that retain the intron.

### Grsf1 binds to Use1 5′UTR and controls Use1 translation

**Figure 4:**
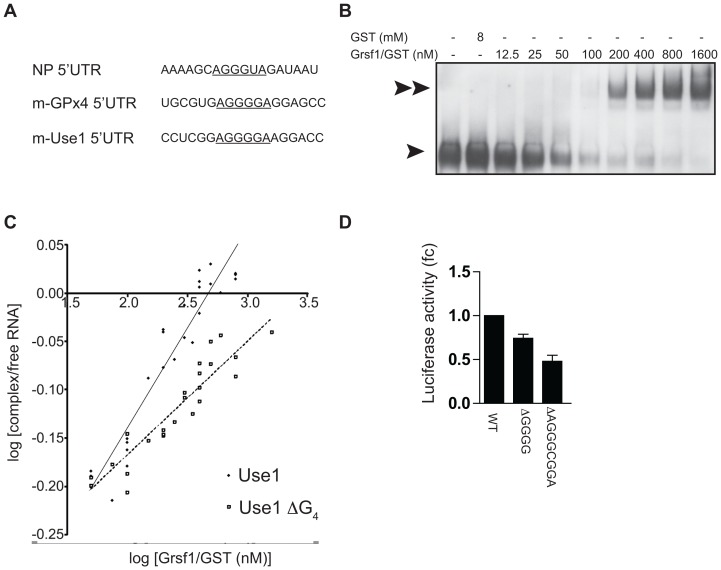
Grsf1 binds to G-rich elements upstream the start codon of the unspliced transcript. (**A**) The A(G)_4_A element of the *NP Influenza* strain, mouse *Gpx4* and mouse *Use1*. (**B**) A digoxigenin labeled RNA fragment of the *Use1* 5′UTR encompassing the A(G)_4_A was incubated with increasing amounts of purified Grsf1-GST as indicated. Northern blots of complexes were stained for digoxigenin to visualize the RNA band-shift signal (1 arrow: free probe; 2 arrows Grsf1-bound probe). (**C**) Quantification of Grsf1-GST binding to the WT RNA probe, or the probe in which the 4 guanidines of the A(G)_4_A element were deleted (Use1 ΔG_4_). The ratio of signal intensities of the shifted complex/free probe was plotted against the Grsf1-GST concentration. The intercept with the X-axis represents the Kd (logarithmic-scale). r^2^(*Use1*) = 90,5, r^2^(*Use1* ΔG4) = 85,4. (**D**) Luciferase activity (fc:fold-change compared to WT) in cell lysates of BaF3 cells, transfected with reporter constructs containing the WT *Use1* 5′UTR, with or without deletions in the G-rich repeats (ΔGGGG or ΔAGGGCGGA). Luciferase activity is corrected for mRNA expression. Error bars indicate standard deviation of 3 experiments.

Analysis of the alternative intron of *Use1* revealed an A(G)_4_A element similar to the Grsf1-binding element found in the 5′UTR of influenza virus and *glutathione peroxidase 4 (Gpx4)* that enhances translation of these transcripts (figure 4A) [30], [43], [51].

The binding of Grsf1 to the G-rich elements in the 5′UTR of *Use1* was analyzed by RNA mobility shift assay. Increasing amounts of *in vitro* synthesized Grsf1 were incubated with a digoxigenin-labeled 40 nt probes containing the G-rich elements (figure 4B), or with a deletion of the A(G)_4_A motif (data not shown). The affinity of Grsf1 to both probes was calculated by plotting the quantified band shift signals against the Grsf1 concentration (figure 4C). This algorithm revealed a linear correlation and the intercept with the X-axis indicated a Kd value of 527 nM for the wild-type probe, and 4.4 µM for the probe with the mutated A(G)_4_A motif, indicating a 8.3-fold difference of affinity dependent on the presence of the A(G)_4_A motif (figure 4C). The Kd for the wt probe is higher than the Kd for Grsf1 binding to the *Gpx4* motif (40 nM), but lower than the Kd of Grsf1 for the Influenza motif A(G)_3_A (1.4 µM) [17], [52].

To test whether the G-rich motives are involved in *Use1* translation regulation, reporter constructs containing the 5′UTR of *Use1* fused to the ORF of the luciferase gene were transfected into Ba/F3 cells. Luciferase activity and luciferase mRNA level were analyzed 24 h post transfection. Deletion of the A(G)_4_A motif reduced luciferase activity to 75%. Comparison of the *Gpx4* and *Use1* 5′UTR indicated that the A(G)_4_A is present in an area with multiple G-repeats, which may form to a secondary structure. This putative structure, as well as the A(G)_4_A sequence may contribute to control of mRNA translation. Therefore, we also deleted the adjacent G-rich element AGGGCGGA, which reduced the luciferase activity to 50% of wt levels (controlled for equal mRNA expression, figure 4D). Thus, G-rich repeats may bind Grsf1 to control mRNA translation of *Use1* reporter constructs. Both a A(G)_4_A and an adjacent A(G)_3_C element may be involved in control of mRNA translation. The activity of the A(G)_3_C motif may explain why the Kd of Grsf1 dropped from 527 nM to 4.4 µM upon deletion of the A(G)_4_A motif, but not to insignificant binding.

### Grsf1 stimulates Use1 translation

**Figure 5:**
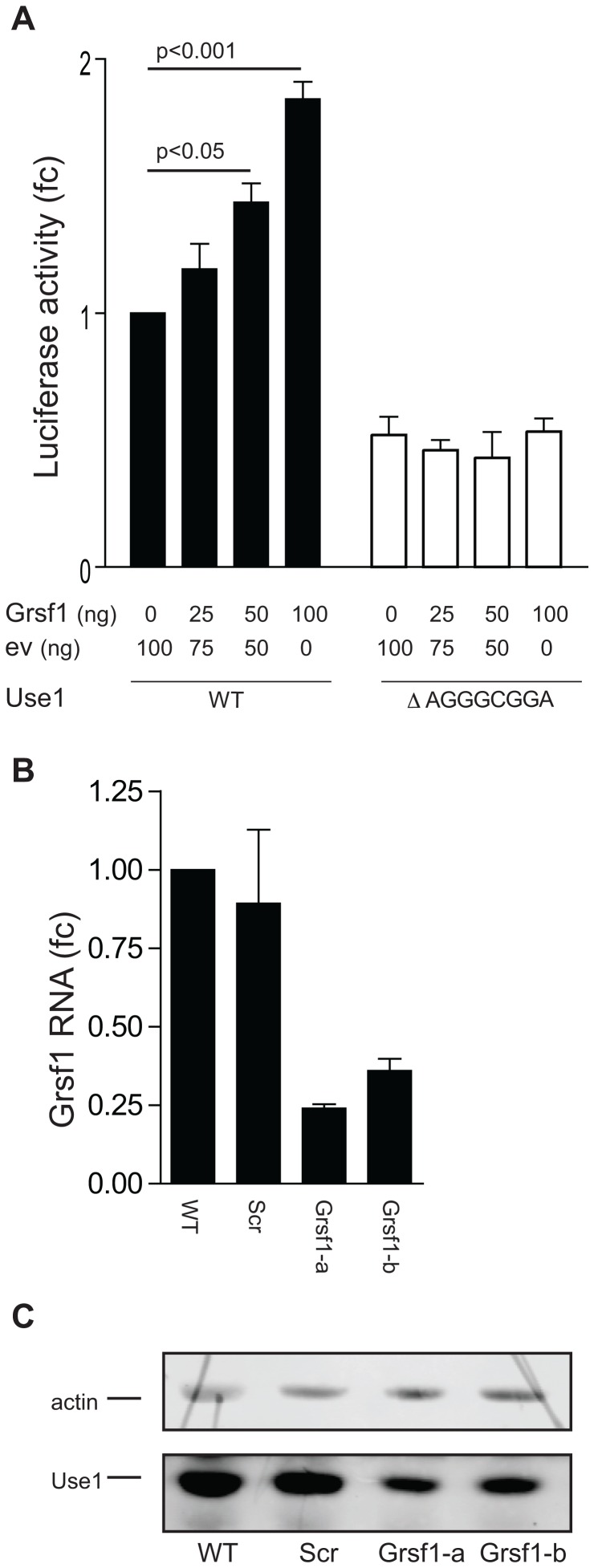
Grsf1 controls translation of Use1. (**A**) Luciferase reporter constructs harboring the *Use1* wt 5′UTR (WT, black bars), or a 5′UTR lacking the AGGGCGGA repeat (open bars), were transfected in NIH3T3 cells together with increasing amounts of a Grsf1 expression plasmid. The total amount of transfected DNA was kept constant by adding additional backbone plasmid DNA (EV, pcDNA3.1). Luciferase activity is corrected for mRNA expression and given as fold-change (fc) compared to WT reporter in absence of Grsf1 overexpression. Error bars indicate standard deviation of 3 experiments. (**B**) I/11cells were transduced with the lentiviral constructs containing two different shRNAs matching *Grsf1* (indicated a and b) or control shRNA (Scr). The efficiency of *Grsf1* transcript knock down was determined using RT-PCR. The expression of *Grsf1* in parental cells is set to 1. Error bars indicate standard deviation of 3 experiments. (**C**) The protein lysates from transduced I/11 cells (representative experiment from B) were tested in WB with anti-actin (42 kDa) and anti-Use1 (30.5 kDa) antibody.

Next we examined whether expression of Grsf1 directly controls Use1 protein expression using over- and underexpression of Grsf1. We first expressed increasing amounts of Grsf1 together with luciferase reporter constructs harboring the WT 5′UTR of *Use1* or a 5′UTR lacking the A(G)_3_ CGGA motif (ΔAGGGCGGA) in NIH3T3 cells. Increasing levels of the Grsf1 expression plasmid, incremented expression of luciferase from the WT construct 2-fold. Luciferase activity from a reporter construct lacking the A(G)_3_ CGGA element was 2-fold reduced compared to the WT construct, and remained unaffected by increasing Grsf1 expression (figure 5A).

Conversely, we also reduced Grsf1 expression using vectors expressing shRNA complementary to Grsf1, or a control shRNA. The mouse I/11 erythroblasts were transduced with the lentiviral constructs and harvested 48 h after puromycin selection. The efficiency of *Grsf1* knock down was measured by quantitative RT-PCR and showed that 2 out of 5 shRNA vectors present in the TRC library [41] resulted in at least 65% knock down (figure 5B). Protein lysates isolated from the mouse erythroblasts were examined by Western blot. Grsf1 knock down clearly repressed expression of Use1 protein (figure 5C). In summary, increased Grsf1 levels induced expression of a reporter for Use1 translation, and Grsf1 knock down reduced Use1 expression in erythroblasts.

### Homology between species

**Figure 6:**
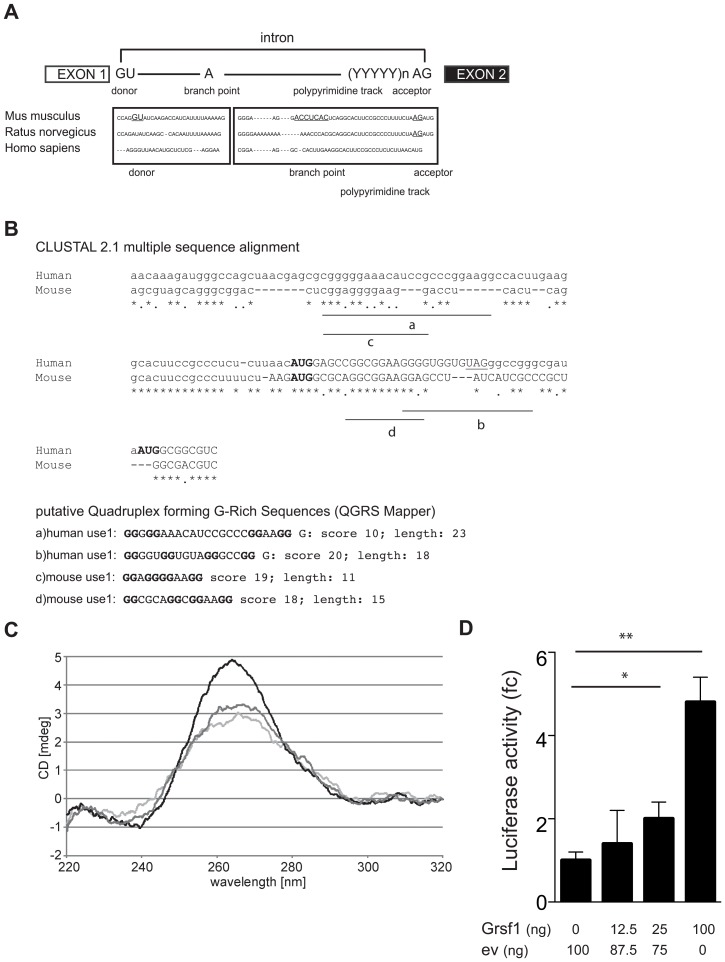
Conservation of the Use1 5′UTR between species. (**A**) Top: a cartoon representing consensus splice sites. Bottom: Alignment of the 5′UTR of *Mus musculus Use1* with *Rattus norvegicus* and *Homo sapiens*. Splice donor and –acceptor sites, and the branching point (A) are indicated by capitals and underlined. (**B**) Top: alignment of the G-rich sequence preceding the AUG start codon of *Use1*. The stopcodon in the human sequence is underlined, asterixes indicate identical nucleotides. Bottom: G-quadruplex structures predicted in the sequences shown in (A) by the QGSRS mapper, including the score and length. (**C**) CD spectroscopy experiments were performed using a 40 nt nucleotide probe of the *Use1* 5′UTR wt sequence (5′-GUA-GCA-GGG-CGG-ACC-UCG-GAG-GGG-AAG-GAC-CUC-ACU-CAG-G-3′), in the presence of 100 mM of KCl (black line), 100 mM NaCl (dark grey line) or in the absence of salts (light grey line). Data were accumulated from ten scans. Presented data are representative for 3 independent experiments. (**D**) The human 5′UTR of *USE1* was cloned in front of the luciferase reporter, and transfected to HEK-293T cells with increasing amounts of Grsf1 expression vector. Luciferase activity is corrected for β-gal expression and given as fold-change (fc) compared to WT reporter in absence of Grsf1 overexpression (n = 3, error bars indicate SD, *p<0.01, **p<0.005).

The splice-donor and -acceptor sites in mouse represent perfect consensus sites. Comparison between species, however, indicated that splicing is not conserved (figure 6A). The splice acceptor site is absent in humans, the splice donor site is absent in rat. Also the branch point A-residue that is present in a perfect consensus in the alternative mouse intron is lacking in humans and rat (figure 6A). Together with the absence of spliced isoforms in EST databases of other species, this strongly suggested that the alternatively spliced intron of mouse could be regarded a *bona fide* part of the *Use1* transcript. Surprisingly, the 5′UTR of primates, man included, shows another unusual feature. The AUG start site of mouse *Use1* is conserved, followed by a stop codon at the 8^th^ triplet, and the AUG start site of the main open reading frame of *USE1* 12 nt downstream of this stop codon (figure 6B). When we analyzed the presence of G-rich motifs in man and mouse, we found similar G-repeats. These repeats obtain scores of 19 (mouse) and 20 (human) as putative G-quadruplex structures using the QGRS-mapper (http://bioinformatics.ramapo.edu/QGRS/index.php; figure 6B). Interestingly, the predicted G-quadruplexes were not present in sequences conserved between man and mouse, but their position relative to the start codon of the *Use1* ORF was similar.

To confirm the presence of G-quadruplexes in the *Use1* 5′UTR, we monitored circular dichroism spectra of the 40 nt RNA construct that binds to Grsf1 (figure 6C). The spectra are characterized by a positive peak at 265 nm and a negative peak at 240 nm, which is characteristic for a parallel quadruplex topology [6]. Spectra were assessed in the presence of KCl, NaCl or without salts. The presence of KCl coincided with the strongest (265 nm, 240 nm) peak intensities. CD spectra obtained in the presence of NaCl or without salts exhibited significant changes of the peak intensities. Thus, our data support the in silico data that indicate the presence of a G-quadruplex structure within the *Use1* 5′UTR, and this structure is stabilized by the monovalent K^+^ cation.

The 5′UTR of human *USE1* was cloned in front of a luciferase reporter gene to test whether translation of human *USE1* is also responsive to Grsf1 expression levels. Transfection of the *USE1*-luciferase reporter in HEK-293T cells with an increasing concentration of Grsf1 expression plasmid enhanced luciferase activity (figure 6D). Thus, the presence of a G-rich motif and responsiveness to Grsf1 is conserved between man and mouse despite a different translation start site.

### Grsf1 and Use1 are required for transient amplification of erythroblasts

**Figure 7:**
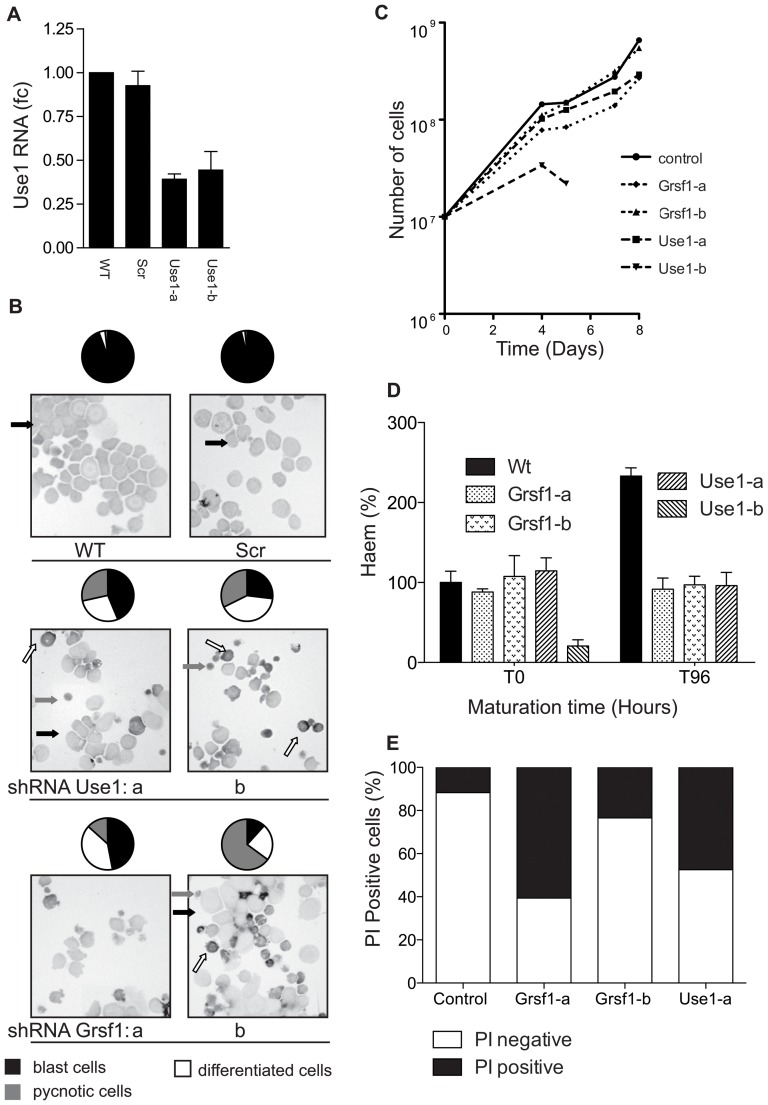
Reduced expression of Use1 or Grsf1 abrogates expansion of erythroblasts. Erythroblasts (I/11 cell line) were transduced with lentiviral shRNA vectors repressing expression of *Use1* (2 distinct sequences, indicated a and b) or *Grsf1* (2 distinct sequences, indicated a and b). Panels show representative data from 1 of 4 replicates. (**A**) *Use1* RNA expression was controlled by RT-PCR 4 days following transfection. For Grsf1 knockdown see figure 4B) (**B**) Cytospins of the cultured cells made 4 days post transduction showed mainly blasts when expressing control shRNA (Scr). Expression of shRNAs matching *Use1* or *Grsf1* increased the number of more mature hemoglobinised cells (Dark staining, white arrows) and pycnotic cells (grey arrows). A quantification of cell types, based on counting >300 cells, is shown above the cytospin as pie-diagram (black, blasts; white, maturing red cells; grey, pycnotic cells). (**C**) shRNA treated cells were counted daily, kept at 2×10^6^/ml and cumulative cell numbers were calculated. (**D**) At the start and 96 hours following induction of differentiation the hemoglobin concentration was measured and calculated as Hb/cell volume in arbitrary units (A.U.). (**E**) At 96 hours the ratio of life/dead cells was determined by staining with propidium iodide (PI). Cells positive for PI were measured by flowcytometry (LSRII, BD).

*Use1* is expressed under conditions that favor amplification of the erythroblast compartment. Upon induction of differentiation to mature erythrocytes, *Use1* is rapidly down regulated [24]. If *Use1* is required for expansion of the erythroblast compartment, its knock down and the knock down of its regulator Grsf1 are expected to abrogate amplification and to induce differentiation. Two lentiviral shRNA vectors able to repress *Grsf1* expression (figure 5B), and two lentiviral shRNA vectors resulting in a 60% knock down of *Use1* expression (figure 7A) were transduced to I/11 mouse erythroblasts. Cytospins were made 4 days following transduction, and revealed a marked increase in small hemoglobinised cells and in pycnotic cells (figure 7B). Cells were calculated and cumulative cell numbers were calculated. Cultures transduction with shRNA *Grsf1-a* and *–b*, or *Use1-a* continued to proliferate, although at reduced rate compared to cells transduced with control shRNA. Cell cultures transduced with shRNA *Use1-b* decreased in cell number and had to be excluded for further analysis of differentiation (figure 7C). Upon induction of differentiation, control cells increased their hemoglobin content, but not upon knockdown of *Use1* or *Grsf1* (figure 7D). Instead cells with suppressed expression of *Use1* or *Grsf1* accumulate dead cells that stain positive for propidium Iodide (PI) (figure 7E). The data shown represent one of four experiments.

## Discussion

Cell proliferation requires increased synthesis of glycosylated lipids and proteins which is dependent on ER-Golgi protein transport by CopII-coated vesicles. Retrograde Golgi to ER transport of chaperones, transport proteins, and other ER resident proteins in CopI vesicles is crucial to maintain ER integrity and allow for anterograde transport to continue. We identified the CopI specific SNARE protein Use1 as a gene under tight translational control in erythroblasts. Translation of *Use1* appeared to be dependent on binding of Grsf1 to a G-repeat present just upstream of the AUG start codon of the open reading frame of *Use1* mRNA. Downregulation of either Grsf1 or Use1 decreased proliferation of erythroblasts.

### Alternative splicing of the mouse Use1 5′ UTR

The 5′RACE experiments indicated that a sequence of 155 nt immediately upstream of the AUG start codon is spliced out in *Use1* transcripts. Reporter assays and polysome analysis indicated that the unspliced transcript is translated preferentially. Alternative splicing of the 5′UTR as a mechanism to expose or remove translational control elements is increasingly recognized as a mechanism that controls protein expression [44], [45]. However, the splice donor or acceptor site are lacking in all other species that were analyzed, suggesting different regulatory mechanisms in these species. In primates the sequence corresponding to the mouse AUG start codon and its surrounding Kozak consensus sequence is identical to the mouse transcript, but after 8 codons it is followed by a stop codon. The start codon of human *USE1* is located 12 nt downstream of this stop codon (a CUG triplet in mouse). This suggests that translation in primates is not dependent on leaky splicing, but on leaky scanning.

### Secondary structures in the 5′UTR of Use1

Several studies identified transcripts that are hypersensitive to mTOR signaling and free eIF4E levels for their translation [24], [26], [33], [49]. These transcripts mostly harbor a complex RNA structure in their 5′UTR [23] and unfolding of such structures is thought to require a higher density of scanning complex loading, together with optimal activity of the eIF4A/B helicase complex. Comparison of luciferase reporter constructs that contain the spliced or unspliced 5′UTR showed that intron sequences inhibit translation in reticulocyte lysate, but enhanced translation in cells. This indicates that secondary structure does not always inhibit translation in cells. In intact cells, secondary structures may have a positive role in translation, and they are most likely recognition sites for RNA binding proteins. The structural element in the alternatively spliced sequence appeared to bind Grsf1. Stable secondary structures with a positive effect on mRNA translation were also found in other transcripts identified as being translationally regulated in erythroblasts [24]. For instance, deletion of the secondary structures in *Nme2* or *Igbp1* enhanced translation in reticulocyte lysate, but impaired translation of reporter constructs in cells [Nieradka et al. unpublished data]. The regulatory domains in the 5′UTR of *Nme2* and *Igbp1*, however, did not bind Grsf1.

### Grsf1 binds a secondary structure upstream the Use1 start codon

The Grsf1 binding site in Influenza and in *Gpx4* was determined to be an A(G)_3_U or A(G)_4_A element respectively whereas a larger structure was suggested to be involved in Grfs1 binding in *Gpx4* [30], [51], [52]. Analysis of the G-rich sequences with the QGRS-mapper that predicts G-quadruplex structures suggests that the A(G)_4_A element is part of such a G-quadruplex structure both in *Use1* and in *Gpx4*, and this prediction was supported by our *in vitro* data obtained by CD spectroscopy. The entire structure was present in the RNA gel shift probes, used to detect specific Grsf1 binding with a Kd intermediate between *Gpx4* and the influenza NP 5′UTR. Binding of Grsf1 to the larger structure, that may be a G-quadruplex, explains why deletion of the nearby A(G)_3_ CGGA element affected translation of the reporter even more than deletion of the A(G)_4_A. Grsf1 potentially binds such structures similar to other hnRNP F/H proteins [51]. Detailed structural data of Grsf1/RNA interactions and the role of G-quadruplex structures are not yet available and more research is needed to obtain such information in future studies.

G-quadruplexes are increasingly recognized as elements that control translation [5], [14]. Interestingly, also transcripts for *TGFβ2* and *CyclinD3* contain G-quadruplex structure [1], [56]. *TGFβ2* is a Epo-induced gene upregulated during erythroid differentiation [32]. When erythroblasts shift from a renewal cell cycle with size control, to differentiation-specific cell cycle control, they shift from CyclinD1 to CyclinD3 expression [40]. It needs to be investigated whether these G-quadruplexes also recruit Grsf1 for the translation of the corresponding mRNA, and whether reduced expression of TGFβ2 and CyclinD3 contributes to impaired differentiation following Grsf1 knockdown.

### Grsf1 is involved in retrograde transport of ER resident proteins

When we reported mEd2 to be translationally regulated [24] it was not yet known to be the human homologue of *S. cerevisiae* Use1p and part of the syntaxin 18 SNARE complex [54]. The essential role of Use1 in control of retrograde transport raises the question whether other regulatory proteins involved in ER-Golgi transport are tightly regulated by Grsf1. SCF controls translation of the ER export receptor *Cnih1* (*Cornichon*) involved in transport of transmembrane proteins and EGF-like ligands (*Gurken* in Drosophila) [18], [19], [24]. The transcripts that required SCF-induced signal transduction to be translated also included the chaperone proteins Hspe1 (hsp10), hspcb (hsp90B) and hspa8 (hsp70 protein a8) [24]. Analysis of their 5′UTR revealed putative secondary structures containing G-rich repeats that fulfill the criteria set for Grsf1 binding sites [30], [51]. Thus, Grsf1 may be involved in translational control of critical ER-resident proteins that may be critical to cope with increased protein and lipid production in proliferating cells.

### Grsf1 expression is transcriptionally and translationally controlled

*Grsf1* is itself translationally regulated. Activation of mTOR results in translation of the RNA binding protein Dazl (Deleted in azoospermia-like), and Dazl subsequently binds the 3′UTR of *Grsf1* to enhance translation [27], [47]. In addition, *Grsf1* transcription is a downstream target of the Wnt/β-catenin pathway. Knock down of *Grsf1* impairs embryonic development and recapitulates Wnt signaling defects [37]. Preliminary data indicate that the Wnt pathway sustains amplification of erythroblasts and induces *Grsf1* expression in primary fetal liver derived erythroblast culture [Nieradka et al. unpublished data].

## Supporting Information

Table S1 List of primers.
(RTF)

Table S2 List of shRNA sequences against Use1, Grsf1 and control sequence. The shRNA sequences were obtained from Sigma's MISSION TRC-Mm 1.0 (Mouse) shRNA library.
(RTF)

## References

[1] Agarwala P, Pandey S, Mapa K, Maiti S (2013) The G-quadruplex augments translation in the 5′ untranslated region of transforming growth factor beta2. Biochemistry 52 (9) 1528–1538. 23387555 10.1021/bi301365g

[2] Antonicka H, Sasarman F, Nishimura T, Paupe V, Shoubridge EA (2013) The mitochondrial RNA-binding protein GRSF1 localizes to RNA granules and is required for posttranscriptional mitochondrial gene expression. Cell metabolism 17 (3) 386–398. 23473033 10.1016/j.cmet.2013.02.006

[3] Bakker WJ, Blazquez-Domingo M, Kolbus A, Besooyen J, Steinlein P, et al (2004) FoxO3a regulates erythroid differentiation and induces BTG1, an activator of protein arginine methyl transferase 1. J Cell Biol 164 (2) 175–184. 14734530 10.1083/jcb.200307056 PMC2172323

[4] Bauer A, Tronche F, Wessely O, Kellendonk C, Reichardt HM, et al (1999) The glucocorticoid receptor is required for stress erythropoiesis. Genes Dev 13 (22) 2996–3002. 10580006 10.1101/gad.13.22.2996 PMC317156

[5] Beaudoin JD, Perreault JP (2010) 5′-UTR G-quadruplex structures acting as translational repressors. Nucleic Acids Res 38 (20) 7022–7036. 20571090 10.1093/nar/gkq557 PMC2978341

[6] Beaudoin JD, Jodoin R, Perreault JP (2014) New scoring system to identify RNA G-quadruplex folding. Nucleic Acids Res 42 (2) 1209–1223. 24121682 10.1093/nar/gkt904 PMC3902908

[7] Beghini A, Peterlongo P, Ripamonti CB, Larizza L, Cairoli R, et al (2000) C-kit mutations in core binding factor leukemias. Blood 95 (2) 726–727. 10660321

[8] Belgareh-Touze N, Corral-Debrinski M, Launhardt H, Galan JM, Munder T, et al (2003) Yeast functional analysis: identification of two essential genes involved in ER to Golgi trafficking. Traffic 4 (9) 607–617. 12911815 10.1034/j.1600-0854.2003.00116.x

[9] Bi X, Corpina RA, Goldberg J (2002) Structure of the Sec23/24-Sar1 pre-budding complex of the COPII vesicle coat. Nature 419 (6904) 271–277. 12239560 10.1038/nature01040

[10] Blazquez-Domingo M, Grech G, von Lindern M (2005) Translation initiation factor 4E inhibits differentiation of erythroid progenitors. Mol Cell Biol 25 (19) 8496–8506. 16166632 10.1128/MCB.25.19.8496-8506.2005 PMC1265736

[11] Bonifacino JS, Glick BS (2004) The mechanisms of vesicle budding and fusion. Cell 116 (2) 153–166. 14744428 10.1016/s0092-8674(03)01079-1

[12] Broudy VC, Lin NL, Priestley GV, Nocka K, Wolf NS (1996) Interaction of stem cell factor and its receptor c-kit mediates lodgment and acute expansion of hematopoietic cells in the murine spleen. Blood 88 (1) 75–81. 8704204

[13] Broxmeyer HE, Cooper S, Lu L, Hangoc G, Anderson D, et al (1991) Effect of murine mast cell growth factor (c-kit proto-oncogene ligand) on colony formation by human marrow hematopoietic progenitor cells. Blood 77 (10) 2142–2149. 1709371

[14] Bugaut A, Balasubramanian S (2012) 5′-UTR RNA G-quadruplexes: translation regulation and targeting. Nucleic Acids Res 40 (11) 4727–4741. 22351747 10.1093/nar/gks068 PMC3367173

[15] Buratti E, Baralle FE (2004) Influence of RNA secondary structure on the pre-mRNA splicing process. Mol Cell Biol 24 (24) 10505–10514. 15572659 10.1128/MCB.24.24.10505-10514.2004 PMC533984

[16] Burri L, Varlamov O, Doege CA, Hofmann K, Beilharz T, et al (2003) A SNARE required for retrograde transport to the endoplasmic reticulum. Proc Natl Acad Sci U S A 100 (17) 9873–9877. 12893879 10.1073/pnas.1734000100 PMC187870

[17] Cassetti MC, Noah DL, Montelione GT, Krug RM (2001) Efficient translation of mRNAs in influenza A virus-infected cells is independent of the viral 5′ untranslated region. Virology 289 (2) 180–185. 11689040 10.1006/viro.2001.1149

[18] Castillon GA, Watanabe R, Taylor M, Schwabe TM, Riezman H (2009) Concentration of GPI-anchored proteins upon ER exit in yeast. Traffic 10 (2) 186–200. 19054390 10.1111/j.1600-0854.2008.00857.x

[19] Castro CP, Piscopo D, Nakagawa T, Derynck R (2007) Cornichon regulates transport and secretion of TGFalpha-related proteins in metazoan cells. J Cell Sci 120 (Pt 14) 2454–2466. 17607000 10.1242/jcs.004200

[20] del Prete MJ, Vernal R, Dolznig H, Mullner EW, Garcia-Sanz JA (2007) Isolation of polysome-bound mRNA from solid tissues amenable for RT-PCR and profiling experiments. Rna 13 (3) 414–421. 17237355 10.1261/rna.79407 PMC1800518

[21] Dilcher M, Veith B, Chidambaram S, Hartmann E, Schmitt HD, et al (2003) Use1p is a yeast SNARE protein required for retrograde traffic to the ER. Embo J 22 (14) 3664–3674. 12853481 10.1093/emboj/cdg339 PMC165609

[22] Dolznig H, Boulme F, Stangl K, Deiner EM, Mikulits W, et al (2001) Establishment of normal, terminally differentiating mouse erythroid progenitors: molecular characterization by cDNA arrays. Faseb J 15 (8) 1442–1444. 11387251 10.1096/fj.00-0705fje

[23] Graff JR, Zimmer SG (2003) Translational control and metastatic progression: enhanced activity of the mRNA cap-binding protein eIF-4E selectively enhances translation of metastasis-related mRNAs. Clin Exp Metastasis 20 (3) 265–273. 12741684 10.1023/a:1022943419011

[24] Grech G, Blazquez-Domingo M, Kolbus A, Bakker WJ, Mullner EW, et al (2008) Igbp1 is part of a positive feedback loop in stem cell factor-dependent, selective mRNA translation initiation inhibiting erythroid differentiation. Blood 112 (7) 2750–2760. 18625885 10.1182/blood-2008-01-133140

[25] Iolascon A, Russo R, Esposito MR, Asci R, Piscopo C, et al (2009) Molecular Analysis of Forty Two Cda Ii Patients: New Mutations in the Sec23b Gene. Search for a Genotype-Phenotype Relationship. Haematologica 10.3324/haematol.2009.014985 PMC2864375 20015893

[26] Jefferies HB, Fumagalli S, Dennis PB, Reinhard C, Pearson RB, et al (1997) Rapamycin suppresses 5′TOP mRNA translation through inhibition of p70s6k. Embo J 16 (12) 3693–3704. 9218810 10.1093/emboj/16.12.3693 PMC1169993

[27] Jiao X, Trifillis P, Kiledjian M (2002) Identification of target messenger RNA substrates for the murine deleted in azoospermia-like RNA-binding protein. Biol Reprod 66 (2) 475–485. 11804965 10.1095/biolreprod66.2.475

[28] Joosten M, Blazquez-Domingo M, Lindeboom F, Boulme F, Van Hoven-Beijen A, et al (2004) Translational control of putative protooncogene Nm23-M2 by cytokines via phosphoinositide 3-kinase signaling. J Biol Chem 279 (37) 38169–38176. 15247270 10.1074/jbc.M401283200

[29] Jourdain AA, Koppen M, Wydro M, Rodley CD, Lightowlers RN, et al (2013) GRSF1 regulates RNA processing in mitochondrial RNA granules. Cell metabolism 17 (3) 399–410. 23473034 10.1016/j.cmet.2013.02.005 PMC3593211

[30] Kash JC, Cunningham DM, Smit MW, Park Y, Fritz D, et al (2002) Selective translation of eukaryotic mRNAs: functional molecular analysis of GRSF-1, a positive regulator of influenza virus protein synthesis. J Virol 76 (20) 10417–10426. 12239318 10.1128/JVI.76.20.10417-10426.2002 PMC136571

[31] Kim TW, Lee H, Kang YK, Choe MS, Ryu MH, et al (2004) Prognostic significance of c-kit mutation in localized gastrointestinal stromal tumors. Clin Cancer Res 10 (9) 3076–3081. 15131046 10.1158/1078-0432.ccr-03-0581

[32] Kolbus A, Blazquez-Domingo M, Carotta S, Bakker W, Luedemann S, et al (2003) Cooperative signaling between cytokine receptors and the glucocorticoid receptor in the expansion of erythroid progenitors: molecular analysis by expression profiling. Blood 102 (9) 3136–3146. 12869505 10.1182/blood-2003-03-0923

[33] Koromilas AE, Lazaris-Karatzas A, Sonenberg N (1992) mRNAs containing extensive secondary structure in their 5′ non-coding region translate efficiently in cells overexpressing initiation factor eIF-4E. Embo J 11 (11) 4153–4158. 1396596 10.1002/j.1460-2075.1992.tb05508.x PMC556925

[34] Lee MC, Miller EA, Goldberg J, Orci L, Schekman R (2004) Bi-directional protein transport between the ER and Golgi. Annu Rev Cell Dev Biol 20: 87–123. 15473836 10.1146/annurev.cellbio.20.010403.105307

[35] Li Y, Jianga Z, Chen H, Ma WJ (2004) A modified quantitative EMSA and its application in the study of RNA–protein interactions. J Biochem Biophys Methods 60: 85–96. 15262445 10.1016/j.jbbm.2004.03.008

[36] Li Y, Zhao X (2013) Fragile X proteins in stem cell maintenance and differentiation. Stem cells (Dayton, Ohio) 10.1002/stem.1698 PMC4255947 24648324

[37] Lickert H, Cox B, Wehrle C, Taketo MM, Kemler R, et al (2005) Dissecting Wnt/beta-catenin signaling during gastrulation using RNA interference in mouse embryos. Development 132 (11) 2599–2609. 15857914 10.1242/dev.01842

[38] Looijenga LH, de Leeuw H, van Oorschot M, van Gurp RJ, Stoop H, et al (2003) Stem cell factor receptor (c-KIT) codon 816 mutations predict development of bilateral testicular germ-cell tumors. Cancer Res 63 (22) 7674–7678. 14633689

[39] Matsuda A, Suzuki Y, Honda G, Muramatsu S, Matsuzaki O, et al (2003) Large-scale identification and characterization of human genes that activate NF-kappaB and MAPK signaling pathways. Oncogene 22 (21) 3307–3318. 12761501 10.1038/sj.onc.1206406

[40] Mikulits W, Dolznig H, Edelmann H, Sauer T, Deiner EM, et al (1997) Dynamics of cell cycle regulators: artifact-free analysis by recultivation of cells synchronized by centrifugal elutriation. DNA Cell Biol 16 (7) 849–859. 9260928 10.1089/dna.1997.16.849

[41] Moffat J, Grueneberg DA, Yang X, Kim SY, Kloepfer AM, et al (2006) A lentiviral RNAi library for human and mouse genes applied to an arrayed viral high-content screen. Cell 124 (6) 1283–1298. 16564017 10.1016/j.cell.2006.01.040

[42] Okumura AJ, Hatsuzawa K, Tamura T, Nagaya H, Saeki K, et al (2006) Involvement of a novel Q-SNARE, D12, in quality control of the endomembrane system. J Biol Chem 281 (7) 4495–4506. 16354670 10.1074/jbc.M509715200

[43] Park YW, Wilusz J, Katze MG (1999) Regulation of eukaryotic protein synthesis: selective influenza viral mRNA translation is mediated by the cellular RNA-binding protein GRSF-1. Proc Natl Acad Sci U S A 96 (12) 6694–6699. 10359774 10.1073/pnas.96.12.6694 PMC21977

[44] Rahim G, Araud T, Jaquier-Gubler P, Curran J (2012) Alternative splicing within the elk-1 5′ untranslated region serves to modulate initiation events downstream of the highly conserved upstream open reading frame 2. Mol Cell Biol 32 (9) 1745–1756. 22354998 10.1128/MCB.06751-11 PMC3347237

[45] Resch AM, Ogurtsov AY, Rogozin IB, Shabalina SA, Koonin EV (2009) Evolution of alternative and constitutive regions of mammalian 5′UTRs. BMC Genomics 10: 162. 19371439 10.1186/1471-2164-10-162 PMC2674463

[46] Ryder SP, Recht MI, Williamson JR (2008) Quantitative analysis of protein-RNA interactions by gel mobility shift. Methods Mol Biol 488: 99–115. 18982286 10.1007/978-1-60327-475-3_7 PMC2928675

[47] Sampath P, Pritchard DK, Pabon L, Reinecke H, Schwartz SM, et al (2008) A hierarchical network controls protein translation during murine embryonic stem cell self-renewal and differentiation. Cell Stem Cell 2 (5) 448–460. 18462695 10.1016/j.stem.2008.03.013

[48] Satchwell TJ, Pellegrin S, Bianchi P, Hawley BR, Gampel A, et al (2013) Characteristic phenotypes associated with congenital dyserythropoietic anemia (type II) manifest at different stages of erythropoiesis. Haematologica 98 (11) 1788–1796. 23935019 10.3324/haematol.2013.085522 PMC3815181

[49] Thoreen CC, Chantranupong L, Keys HR, Wang T, Gray NS, et al (2012) A unifying model for mTORC1-mediated regulation of mRNA translation. Nature 485 (7396) 109–113. 22552098 10.1038/nature11083 PMC3347774

[50] Uemura T, Sato T, Aoki T, Yamamoto A, Okada T, et al (2009) p31 deficiency influences endoplasmic reticulum tubular morphology and cell survival. Mol Cell Biol 29 (7) 1869–1881. 19188447 10.1128/MCB.01089-08 PMC2655616

[51] Ufer C (2012) The biology of the RNA binding protein guanine-rich sequence binding factor 1. Curr Protein Pept Sci 13 (4) 347–357. 22708492 10.2174/138920312801619457

[52] Ufer C, Wang CC, Fahling M, Schiebel H, Thiele BJ, et al (2008) Translational regulation of glutathione peroxidase 4 expression through guanine-rich sequence-binding factor 1 is essential for embryonic brain development. Genes Dev 22 (13) 1838–1850. 18593884 10.1101/gad.466308 PMC2492670

[53] van Dijk TB, van Den Akker E, Amelsvoort MP, Mano H, Lowenberg B, et al (2000) Stem cell factor induces phosphatidylinositol 3′-kinase-dependent Lyn/Tec/Dok-1 complex formation in hematopoietic cells. Blood 96 (10) 3406–3413. 11071635

[54] Verrier SE, Willmann M, Wenzel D, Winter U, von Mollard GF, et al (2008) Members of a mammalian SNARE complex interact in the endoplasmic reticulum in vivo and are found in COPI vesicles. European journal of cell biology 87 (11) 863–878. 18834646 10.1016/j.ejcb.2008.07.003

[55] von Lindern M, Deiner EM, Dolznig H, Parren-Van Amelsvoort M, Hayman MJ, et al (2001) Leukemic transformation of normal murine erythroid progenitors: v- and c-ErbB act through signaling pathways activated by the EpoR and c-Kit in stress erythropoiesis. Oncogene 20 (28) 3651–3664. 11439328 10.1038/sj.onc.1204494

[56] Weng HY, Huang HL, Zhao PP, Zhou H, Qu LH (2012) Translational repression of cyclin D3 by a stable G-quadruplex in its 5′ UTR: implications for cell cycle regulation. RNA Biol 9 (8) 1099–1109. 22858673 10.4161/rna.21210 PMC3551864

[57] Zheng R, Klang K, Gorin NC, Small D (2004) Lack of KIT or FMS internal tandem duplications but co-expression with ligands in AML. Leuk Res 28 (2) 121–126. 14654075 10.1016/s0145-2126(03)00184-x

[58] Zhong XY, Wang P, Han J, Rosenfeld MG, Fu XD (2009) SR proteins in vertical integration of gene expression from transcription to RNA processing to translation. Mol Cell 35 (1) 1–10. 19595711 10.1016/j.molcel.2009.06.016 PMC2744344

