# Supplementary material for: Grsf1-Induced Translation of the SNARE Protein Use1 Is Required for Expansion of the Erythroid Compartment
Source: PLoS One. 2014 Sep 3;9(9):e104631. doi: 10.1371/journal.pone.0104631 (PMC4153549; doi:10.1371/journal.pone.0104631)
Supplement: Table S1 — List of primers. (RTF) [file pone.0104631.s001.rtf]

PRIMERS	
NAME	FORWARD (5'-3')	REVERSE (5'-3')	
Primers used in RACE assay	
GSP1		CACCTCACATTCTCCAG	
GSP2		TCACAGCGACAAAGCAGC	
GSP3		TGAAGCAGAGTCTCTGTAGTCC	
Primer used in Sequencing	
M13	GTAAAACGACGGCCAGT		
Primers used in RT-PCR	
RI	TCCAGTGTGAGCAGCTGAG	TGCAGGCACTGAAGCACCA	
a-tubulin	GGCACCGGCTCTGGCTTCAC	GCACGCTGCACCTTGGCCAG	
GAPDH	CCATCACCATCTTCCAGGAGCGA	GGATGACCTTGCCCACAGCCTTG	
Grsf1	GAATCCAAAACTACCTACCTGGAAG	CAGCTGTAAGGAAGTCCTCTCAG	
Use1 total	TCAGCATCCGAGCTAGACC	TCACTGACTTCTGTGCATGC	
Use1 intron	TCTACTTCCGTCCCAAGC	TCACAGCGACAAAGCAGC	
Intron/luciferase	AGAGACAGGGACGAAAACGA	CTTATGCAGTTGCTCTCC	
Luciferase	TCAGATTCTCGCATGCCAG	TGGTACTAGCAACGCAC	
Use1 5'UTR probe used for the RNA mobility shift assay	
Remsa probe	CATCTTAGAAAAGGGCGGAAGTG	AATTTAATACGACTCACTATAGGAACTCTACTTCCGTCCCAAGCG	
Use1 constructs used for the reporter assay	
WT/In	CCGCTCGAGCGGTTAATACGACTCACTATAGGGCAGGTCAAGGTTCCCGCTACAG	CATGCCATGGCCTTCAGAAAGTCCACTTTG	
- globin PCR 1	CCGCTCGAGCGGTTAATACGACTCACTATAGGGCAGGTCAAGGTTCCCGCTACAG	tcccatagactcacctggccttttcgctttttgaagc	
- globin PCR 2	TTCAAAAAGCGAAAAAGGCCAGGTGAGTCTATGGGACG	TAGGCTCCTTCCGCCTGCGCCATCTGTGGGAGGAAGATAAGAGS	
- globin PCR 3	TACCTCTTATCTTCCTCCCACAGATGGCGCAGGCGG	CATGCCATGGCCTTCAGAAAGTCCACTTTG      	
SS SDM	CGAAAAAGGCCAAGTATCAAGACCATC	GATGGTCTTGATACTTGGCCTTTTTCG	
GGGG SDM	CAGGGCGGACCTCGGAGGACCTCACTCAGG	CCTGAGTGAGGTCCTCCGAGGTCCGCCCTG	
AGGGCGGA SDM	CGTCCCAAGCGTAGCCCTCGGAGGGGAAGGA	TCCTTCCCCTCCGAGGGCTACGCTTGGGACG	
Human WT	GCTCGAG AGCAATCCTT TCCCACCTCT	CG CCATGG TCGCCCGGCC CTACACCA	

Table S1

*) Single Direct Mutagenesis


 
