# Supplementary material for: Grsf1-Induced Translation of the SNARE Protein Use1 Is Required for Expansion of the Erythroid Compartment
Source: PLoS One. 2014 Sep 3;9(9):e104631. doi: 10.1371/journal.pone.0104631 (PMC4153549; doi:10.1371/journal.pone.0104631)
Supplement: Table S2 — List of shRNA sequences against Use1, Grsf1 and control sequence. The shRNA sequences were obtained from Sigma's MISSION TRC-Mm 1.0 (Mouse) shRNA library. (RTF) [file pone.0104631.s002.rtf]

Table S2


	
NAME	TARGET	SEQUENCE	
shRNA (1849)  a Grsf1
TRCN0000109075	NM_178700.2	CCGGCCAGTCTGATTTGGTCAAATACTCGAGTATTTGACCAAATCAGACTGGTTTTTG	
shRNA (1850)  b Grsf1
TRCN0000109076	NM_178700.2	CCGGCCTCCTAAATAGAGATGGGAACTCGAGTTCCCATCTCTATTTAGGAGGTTTTTG	
shRNA (1119)  a Use1
TRCN0000126244	NM_025917.1	CCGGCCTGTCACATTCACTCAAGATCTCGAGATCTTGAGTGAATGTGACAGGTTTTTG	
shRNA (1121)  b Use1
TRCN0000126246	NM_025917.1	CCGGCCAGAGTGTCATCAAGAAGGACTCGAGTCCTTCTTGATGACACTCTGGTTTTTG	
shRNA  SCR
SHC002		CAACAAGATGAAGAGCACCAA	
